# Surgical Excision of an Adrenal Neuroblastoma in a Dog

**DOI:** 10.3389/fvets.2019.00161

**Published:** 2019-05-29

**Authors:** Lisa Alexandra Mestrinho, Maria Inês Marques, Sandra Jesus, Hugo Pissarra, Maria Peleteiro, Antonio Ferreira

**Affiliations:** CIISA - Centre for Interdisciplinary Research in Animal Health, Faculty of Veterinary Medicine, University of Lisbon, Lisbon, Portugal

**Keywords:** neuroblastoma, adrenal gland, embryonal tumor, surgery, dog

## Abstract

A 11-month-old, intact male, Rhodesian Ridgeback was presented to the Veterinary Teaching Hospital with signs of inappetence, lethargy, and abdominal pain for 3 days. A large and well-defined abdominal retroperitoneal mass, related with the left kidney, at the expected location of the adrenal gland, was revealed by radiography, ultrasound, and computed tomography. The mass extended caudally to the iliac artery bifurcation, compressing the aorta, caudal vena cava, and both kidneys. Cytology findings were compatible with a malignant round cell tumor. The most probable diagnosis was neuroblastoma. Following a comprehensive discussion with the owners about a treatment plan, surgical excision was performed. Because a wide excision would compromise major vessels, excision was performed after careful dissection of the aorta and vena cava. The left kidney was removed because the proximal ureter could not be separated from the tumor. The animal recovered successfully. Diagnosis was confirmed by histopathology and immunohistochemistry, but the owners decided not to pursue any further treatment. Clinical signs of abdominal pain recurred within 1 month following surgery. Therefore, the animal was euthanized upon the owners' request. This report describes the diagnosis, surgical treatment, and follow-up of a dog with an abdominal peripheral neuroblastoma.

## Background

Neuroblastomas are tumors of neuroectodermal origin, arising from primitive sympathetic ganglion cells (neural crest) (1). Although peripheral neuroblastomas are rare in dogs, they are an important pediatric cancer in humans (2–8). In most cases, neuroblastomas occur in the abdominal cavity as large masses. Some neuroblastomas have an adrenal origin (2–8) while others have cervical and retropharyngeal origins (4, 8, 9).

To the best of our knowledge, there are no reports on any attempted treatment of peripheral neuroblastomas in veterinary literature. Additionally, prognostic information for neuroblastomas is scarce. In most cases, they were described and studied during diagnostic necropsy.

This case report describes the detailed diagnostic work-up, attempted surgical excision, and short-term follow-up of an adrenal neuroblastoma in a young dog.

## Case presentation

An 11-month-old intact Rhodesian Ridgeback dog was presented to the Veterinary Teaching Hospital of the Faculty of Veterinary Medicine, University of Lisbon. The owners reported clinical signs of inappetence, lethargy, and presumed cervical pain which lasted for 3 days. There was no previous history of other diseases and vaccination and deworming were up-to-date.

Clinical examination revealed slight abdominal pain and a large, palpable, mid-cranial abdominal mass with firm consistency. Cervical pain was not found and the neurological exam results were normal, except for a stilted gait of the animal, possibly due to abdominal pain. Blood samples were collected for hematology and biochemistry (including tests for albumin, total proteins, alanine aminotransferase, alkaline phosphatase, creatinine, blood urea nitrogen, potassium, and calcium) and the results of these tests were within reference range. Abdominal and chest radiography, abdominal ultrasound, and thoraco-abdominal computed tomography (CT) were performed to identify and stage the mass (Supplementary Video 1).

The mass was large and located cranially, causing ventral deviation of the intestine and spleen. No chest anomalies were identified on left and right lateral and ventro-dorsal projections. During ultrasonography, we identified a large retroperitoneal mass with irregular margins, located cranial to the left kidney, extending caudo-medially to the iliac artery bifurcation, and compressing the aorta and caudal vena cava. The mass had mixed echogenicity with underlying hypoechoic diffuse background with small scattered echogenic foci, possibly due to calcification. No thrombi of major vessels were identified. The left and right kidneys showed discrete pyelic dilations of 0.7 cm and 0.5 cm in diameter, respectively. This was attributed to the compressive effect of the mass. The liver and spleen appeared normal during ultrasonography. Fine needle aspiration was performed during the abdominal ultrasound. The cytological diagnosis was a malignant non-differentiated neoplasia, due to an abundant population of loosely arranged round to oval cells with marked anisocytosis and anisokaryosis. Nuclear indentations and multiple nucleoli were commonly seen.

**Figure 1:**
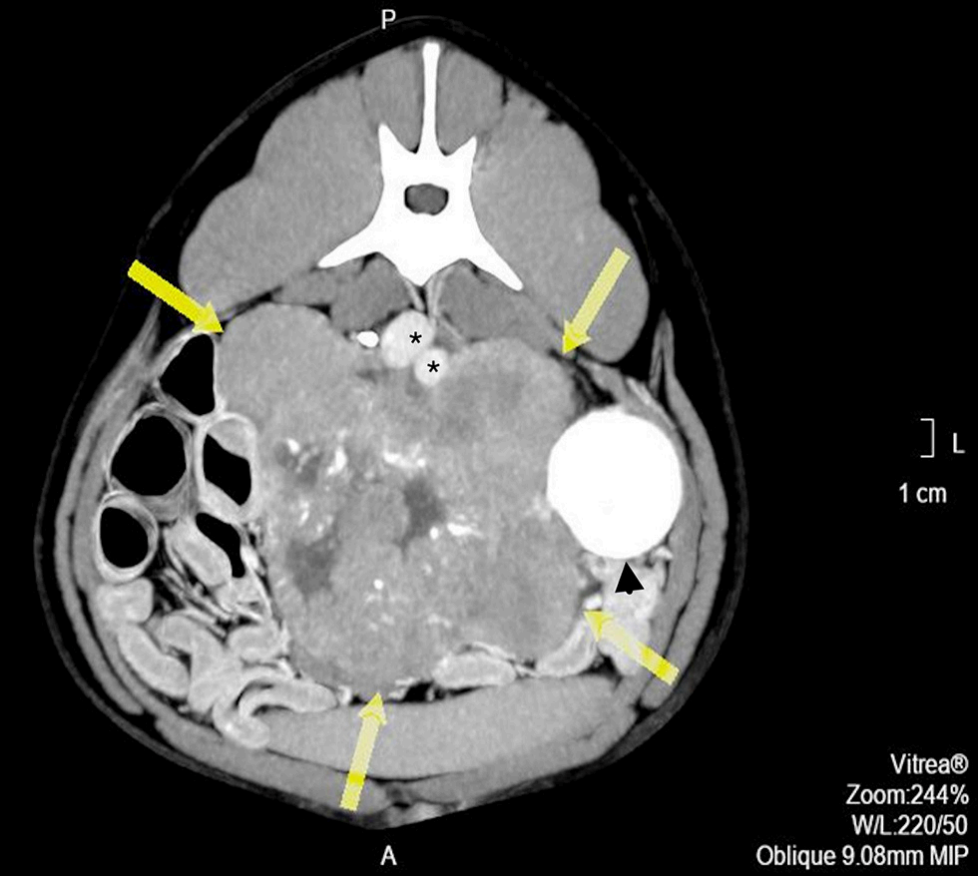
Pre-operative contrast enhanced computed tomography axial image showing a heterogeneous mass, with hypo-attenuating areas corresponding to necrosis or liquid intermixed with stippled calcifications (arrows). *indicates major vessels.

**Figure 2:**
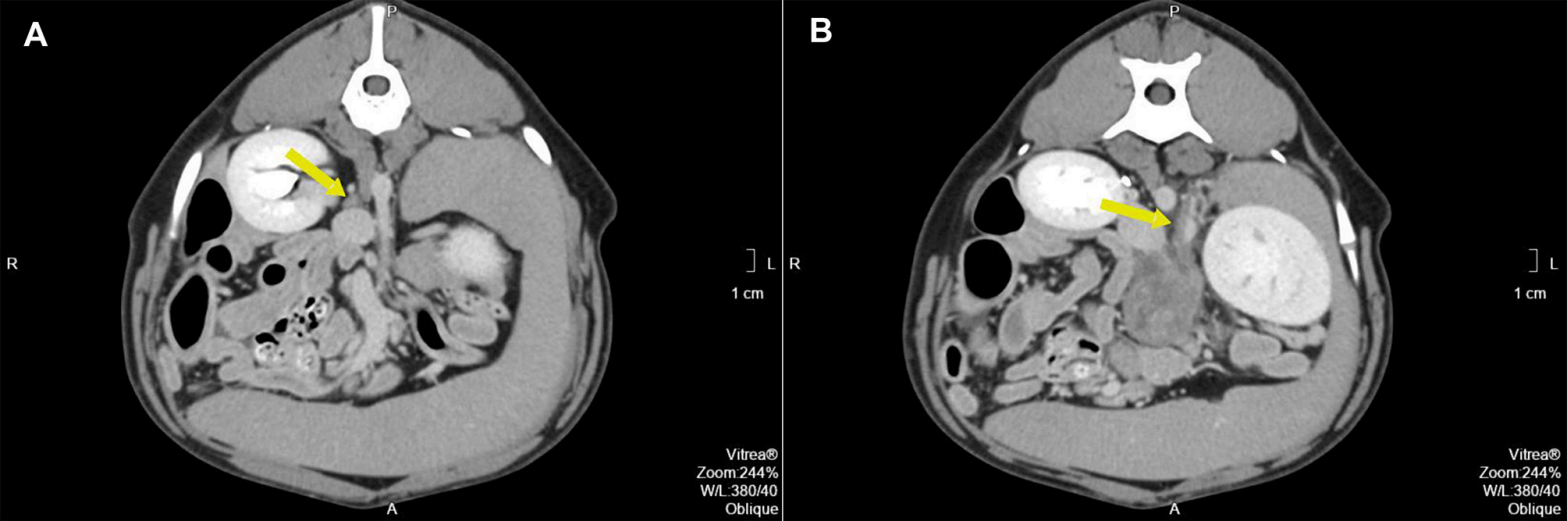
**(A)** Pre-operative contrast enhanced computed tomography axial image of the right adrenal gland (arrow) with the corresponding phrenicoabdominal vein. **(B)** Pre-operative contrast enhanced computed tomography axial image of the expected location of the left adrenal gland (arrow). The arrow points to the left phrenicoabdominal vein. The mass extends ventrally.

**Figure 3:**
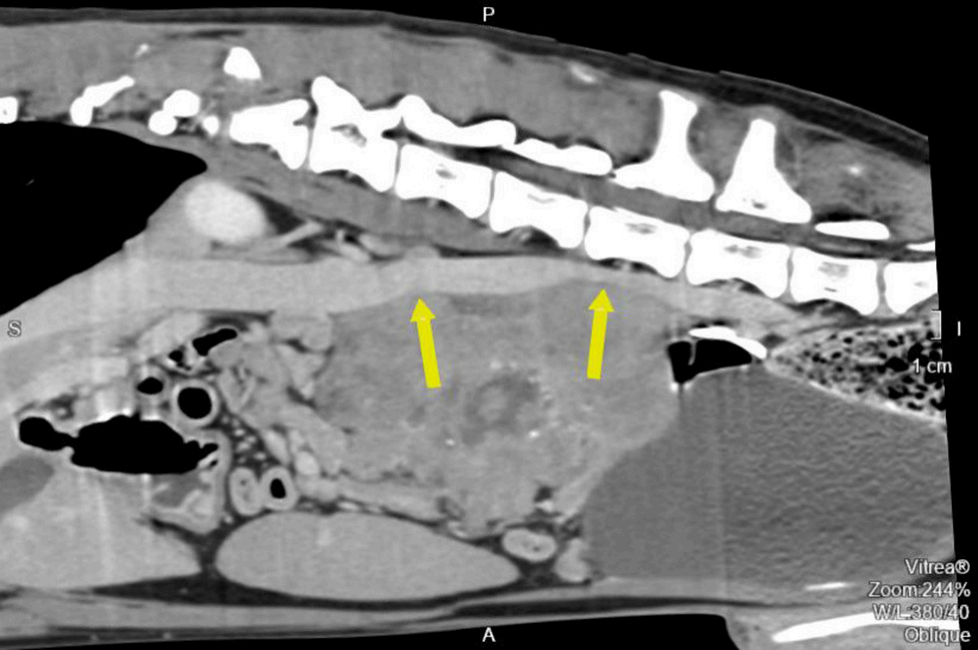
Pre-operative contrast enhanced computed tomography reconstructed oblique sagittal image along the cava vein. The arrow also points to the two compression points.

Pre- and post-contrast CT scans were performed using a Toshiba Astellion (16 slice-light speed) machine. Images were obtained using the helicoidal acquisition mode in a single phase, with 10-mm slices and reconstruction at 1 mm for the abdomen and 3 mm slices and reconstruction at 1 mm for the thorax, at 120 KV and 300 mA. The contrast medium was Ioxitalamate sodium (Telebrix 35, Guerbet, 2 ml/Kg). Analysis of the abdominal series was performed using the soft tissue algorithm (W 360, L 60) and that of the thorax was performed using the lung (W 1600, L400) and soft tissue (W 360, L 60) algorithms. Pre-contrast CT revealed a large lobulated (120.7 × 123.4 × 84.3 mm), heterogeneous soft tissue density mass, with irregular margins and mineralized foci, of unknown origin, located in the retroperitoneal space, extending from the level of the second to the sixth lumbar vertebrae. On the post-contrast study, the mass showed a moderate mixed contrast uptake pattern, with hypointense central areas, suggestive of necrosis or fluid intermixed with stippled calcifications (Figure 1). Slight left renomegaly and pyelectasis were present. The right ureter, adrenal gland, and corresponding phrenicoabdominal vein were clearly identifiable (Figure 2A). The left phrenicoabdominal vein was identified whereas the left adrenal gland and left ureter, which was encased in the retroperitoneal mass, were not (Figure 2B). There was extrinsic ventral compression of the vena cava and aorta, with no signs of intraluminal invasion. The compression of the vena cava was the most severe and was observed at two points: the most cranial (26 mm extension) at the L2–L3 level and the second (50 mm extension) at the L4–L5 level, ending 25 mm at the confluence of the iliac veins. Compression led to a reduction of more than 50% of its diameter at the second point (Figure 3). A discrete peritoneal and retroperitoneal effusion was identified dorsal and medial to the left kidney. No abnormal findings were detected on pre- or post-contrast chest images.

The differential diagnoses at this point were lymphoma, adenoma, carcinoma, myelolipoma, pheochromocytoma, neuroblastoma, and ganglioneuroma. The clinical and cytological features of the mass and the young age of the animal were highly suggestive of an adrenal neuroblastoma. This was a large, unique abdominal mass originating in the retroperitoneal space, anatomically related to the left kidney. The left adrenal gland was absent, there were no signs of distant metastasis. Cytology revealed non-differentiated neoplasia (Supplementary Figure 1). Considering these findings, different treatment protocols were discussed with the owners. Initially, we proposed biopsy to confirm diagnosis, followed by multimodal treatment with surgery and chemotherapy. However, the size of the mass and the waiting time from biopsy to treatment was a concern. Excision seemed feasible and was chosen by the owners who accepted the associated risks. Tramadol (Tramadol, Labesfal, approximately 6 mg/kg) was administered twice daily until surgery.

The animal underwent exploratory laparotomy 7 days after initial presentation. Anesthesia was performed according to a multimodal protocol with intra-muscular administration of methadone (Senfortan, Ecuphar, 3 mg/kg) with acepromazine (Calmivet, Vetoquinol, 0.005 mg/kg) as pre-medication, 30 min before induction. An intravenous catheter was placed in the cephalic vein. Induction was performed with intra-venous administration of midazolam (Midazolam, B Braun, 0.2 mg/kg) combined with Propofol (Propovet, Ecuphar) to allow intubation. Ketamine (Clorketam, Vetoquinol, 0.3 mg/kg/h), fentanyl (Fentadon, Dechra, 0.0024 mg/kg/h), and lidocaine (Anesthesin, Medinfar, 1 mg/kg/h) were administered throughout the procedure at a constant rate and during the 12-h post-operative period. Because the procedure was long, antibiotic prophylaxis was performed by administering cefazolin (Cefazolina, Labesfal, 22 mg/kg).

Anesthetic monitoring was performed using a multiparameter monitor with electrocardiography, side stream capnography, pulse oximetry, and oscillometric non-invasive blood pressure and temperature monitoring. Non-invasive blood pressure monitoring was performed using the Doppler method (PetMAP, Ramsey Medical Inc) for accurate measurements.

An abdominal ventral midline incision from the xiphoid to the pubis was performed to gain full access. The mass occupied most of the abdominal cavity. Inspection and palpation of other internal organs did not reveal signs of metastasis. The mass was primary, extensive, lobulated, and separated from peritoneal organs by the parietal peritoneum.

**Figure 4:**
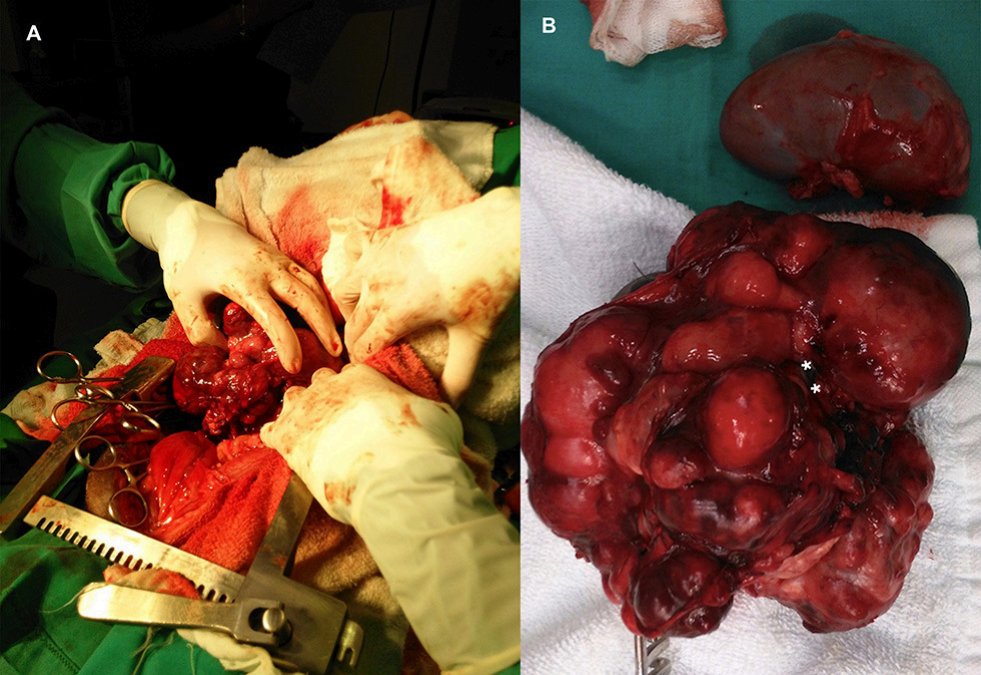
**(A)** Intraoperative image at time of excision of an adrenal neuroblastoma. **(B)** Abdominal neuroblastoma excised together with the kidney. Point of mass attachment to the kidney hilus and ureter (asterisks).

Wide excision was not possible because this would result in resection of the major vessels at the attachment point. Consequently, the tumor was excised using a method previously described in humans (10). This technique relies on the fact that neuroblastomas usually do not invade the tunica adventitia of major blood vessels. The surgical plan included excision of the left kidney with the tumor if surgical dissection of the ureter was not feasible or if significant intraoperative blood loss would occur. Because severe hemorrhage was expected, intraoperative transfusion was also planned. The retroperitoneum was incised to allow a blunt dissection of the tumor from the dorsal abdominal wall. The mass was well-defined but lobulated and heterogeneous, grossly attached to the parietal peritoneum, left kidney, and ureter at the proximal third of the ureter medially (Figure 4A). The surgical procedure was divided into three phases: vessel identification, vessel clearance, and tumor removal. The first phase was performed cranially and caudally to the mass. After vessel identification, moistened, size 2, silk sutures were looped and passed through a short extension tube to facilitate handling and to be used as Rumel tourniquets for hemorrhage control. The second phase included blunt and sharp dissection around the vessels and kidney. The tumor was separated from the blood vessel through a sharp dissection along the subadventitial plane, aided by gentle traction (10). Dissection started cranially to the tumor and then followed the vessels. Since the phases were not distinct and occurred simultaneously, in some instances, silastic tubes were also used for traction and tourniquets as the vessels were being cleared. The tumor was attached to more than 50% of circumference of the vessel, but complete encasement was not observed (Figures 1, 3). Dissection through the artery adventitia was easier compared to dissection through the vena cava, which was accidentally incised. Hemorrhage was controlled with tourniquets and manual compression. The incision was then repaired using a 6/0 poliglecaprone suture in a simple continuous pattern. The tumor was excised in one piece.

Dissection of the left kidney from the mass was possible, but not the ureter. Dissection at the level of the hilus was attempted but was unsuccessful due to the gross anatomic distortion caused by the tumor. Therefore, as planned, a nephrectomy was performed (Figure 4B).

The retroperitoneal space was sutured using a 4/0 poliglecaprone suture in a simple interrupted pattern. Abdominal closure was performed in three layers: 2/0 and 3/0 poliglecaprone sutures were used to close the abdominal fascia, subcutaneous tissue, and skin, respectively. Transfusion was not performed as planned because intraoperative blood loss was approximately 7% of total blood volume and intraoperative blood pressures were maintained during the procedure (11).

Postoperative management included constant infusion for 12 h, fentanyl transdermal patch (Durogesic, Janssen Pharmaceutica, 100 μg), applied at the time of surgery, for 72 h, and meloxicam (Metacan, Boeringer Ingelheim, 0.1 mg/kg) once daily for 3 days. No major complication was identified, and the animal was discharged 3 days after surgery. Oral medication included Firocoxib (Previcox, Merial, 5 mg/kg), administered for 5 days. Mirtazapine (Mirtazapina, Laboratórios Atral, 30 mg) was prescribed once daily from the fourth day post-operation, due to the reduced appetite of the animal. The animal fully recovered 1 week later.

**Figure 5:**
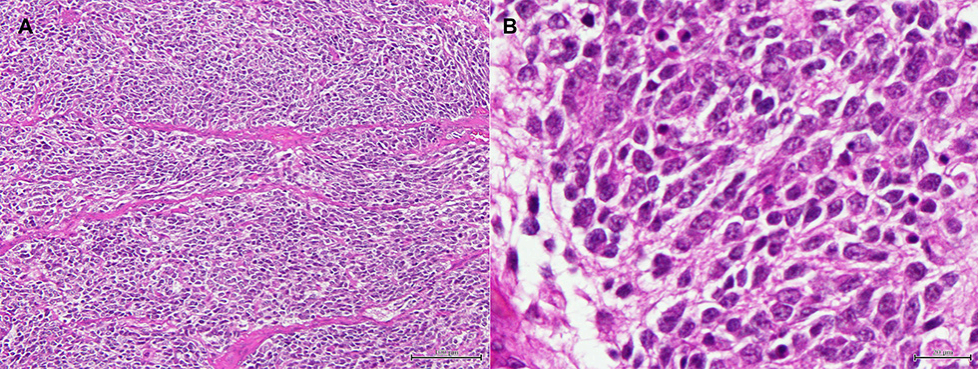
**(A)** Adrenal tumor. Low magnification image showing round to oval cells organized in ill-defined lobules, within a very poor stroma. (H&E, bar = 100 μm). **(B)** Adrenal tumor. High magnification image of the tumor cells. The cell limits are ill-defined, the cytoplasm is scant and pale, and the ovoid-shaped nucleus shows stippled chromatin distribution with no conspicuous nucleolus. (H&E, bar = 20 μm).

Histopathology of the mass confirmed its neoplastic nature. Tumor cells were round to oval, organized in lobules within a poor stroma (Figure 5). Cell limits were ill-defined, cytoplasm was scant and pale, and the ovoid-shaped nucleus showed stippled chromatin distribution with no conspicuous nucleolus. Mitotic index was moderate with six mitoses per ten high power fields. Immunohistochemistry confirmed the neuroendocrine nature of the tumor. In Table 1, details of the immunohistochemistry are shown. In all cases, the DAKO EnVision Detection System, Peroxidase/DAB, Rabbit/Mouse was used. Negative controls were obtained by replacing the primary antibody with phosphate-buffered saline. Tumor cells were positive for neurofilament protein (estimated positivity−10% of the tumor area), neuron specific enolase (NSE), and synaptophysin (SYN) (Supplementary Figures 2–4), and negative for glial fibrillary acidic protein (GFAP), pancytokeratin, lymphoid cell markers CD3 and CD20, and vimentin. A very high division rate, estimated at 68%, was confirmed by Ki67 labeling (Supplementary Figure 5). The final diagnosis was adrenal neuroblastoma.

**Table 1:** Immunohistochemistry details.

	**Manufacturer**	**Type**	**Clone**	**Dilution**	**Incubation time**	**Positive control**
CD20	Biocare	Policlonal		1/200	60 mn	Dog lymph node
CD3	Dako	Policlonal		1/400	60 mn	Dog lymph node
Vimentin	Dako	Monoclonal	V9	1/100	60 mn	Dog colon
CK	Dako	Monoclonal	AE1/AE3	1/400	60 mn	Dog colon
NSE	Novocastra	Monoclonal	NCL-L-NSE2	1/80	60 mn	Dog nerve fiber
Synaptophysin	Roche	Monoclonal	DAK-SYNAP	Ready	60 mn	Dog brain
Neurofilament Protein	Roche	Monoclonal	DF11	Ready	60 mn	Dog brain
Ki67	Dako	Monoclonal	Mib-1	1/100	60 mn	Dog ovary

The adjuvant chemotherapy plan was refused by the owners. Thirty-three days after surgery and 40 days after initial presentation the owners returned reporting similar complaints. During clinical examination, the dog presented a stilted gait, associated with abdominal and lumbosacral pain. The owners refused to pursue any further investigations or treatments. The dog was subsequently euthanized upon their request.

Post mortem examination revealed one enlarged nodule in the cranial mediastinum (60 × 25 mm). The central mesenteric lymph node was also enlarged (75 × 35 mm) together with numerous small hepatic nodules disseminated throughout the parenchyma (average diameter of 5 mm). No signs of macroscopic tumor recurrence were found at the surgical site. Histological examination confirmed the lesions to be metastasis of the adrenal neuroblastoma, similar to the results obtained through immunohistochemistry.

## Discussion

In dogs, most neuroblastomas originating from the adrenal gland are not associated with any specific signalment. Symptoms range from asymptomatic to lethargic, weight loss, abdominal distension, and compression-related signs such as dyspnea, pain, ataxia, paresis, diarrhea, and edema (2, 5–8). Some of these signs were observed in the present case. These tumors usually have reached a considerable size before being detected and the prognosis is poor due to the advanced stage of the disease at the time of presentation (3, 4, 6–9). These tumors are large but often without signs of metastasis at the time of diagnosis, as encountered in this case, with X-ray, abdominal ultrasound, and thoraco-abdominal CT showing no metastatic-related findings.

The biological behavior of this embryonal tumor in the dog resembles what is observed in children (8). To the best of the authors' knowledge, there are no reported attempts to treat abdominal neuroblastoma in a dog. The prognosis presented to the owners was very poor and uncertain. In children, the overall survival rate for neuroblastoma can be very high ranging from 75 to 98% at 5 years depending on the stage of the tumor and treatment strategy (12–14). Each treatment strategy must be evaluated individually, but surgery is the mainstay of treatment for most cases, since the prognosis can be reasonable with surgery alone (15, 16), or improve the outcome in other cases (16, 17). Additionally, adjuvant or neoadjuvant treatments can also be used to reduce mass size when surgical risk is high (15–17). The combined treatment includes surgery, adjuvant or neoadjuvant chemotherapy, and/or radiotherapy (1, 13). For the present case, radiotherapy was unavailable, and the available drugs for chemotherapy included carboplatin, cyclophosphamide, doxorubicin, and etoposide (12). Dosages for these drugs in dogs have been established in the literature.

Surgical excision was attempted as the principal approach to reduce size and increase the chance for chemotherapy control. Since a wide excision was not possible due to adhesion to major vessels, a technique described for treating similar tumors in humans was used (10). In the aforementioned technique, a transverse supra umbilical incision was made to gain access to the abdomen. However, in the present case, a xiphoid-pubic incision was made, which allowed full access to the mass.

Sharp dissection between the adventitia and tunica media of the aorta and caval vein is possible and was successfully done in this case. However, there was one iatrogenic incident which led to rupture of the vena cava, which could be repaired successfully. With security maneuvers such as the use of tourniquets and elastics, the risk of hemorrhage during sharp dissection along major vessels can be controlled. In this case, the predicted intraoperative risks were based on the information provided by the CT and ultrasound, which included acute abdominal hemorrhage and the need to perform a left nephrectomy.

The definitive diagnosis of neuroblastoma in the present case was based on histological features, immunohistochemistry results, and the young age of the dog. Tumors of the adrenal medulla, which arise from neuroectodermal cells of the neural crest, can differentiate into sympathetic nervous system or secretory cells. When the sympathetic nervous system cell line is adopted, the tumors may be very primitive neuroblastomas or more differentiated tumors formed by sympathetic ganglion cells, the ganglioneuromas. Neuroblastomas may also be formed by more primitive neural crest cells that have not differentiated yet (18). When these cells differentiate into secretory cells of the medulla, they become chromaffin-like cells and tumors from these cells are called pheochromocytomas. In distinguishing between the aforementioned tumor types, various aspects need to be considered. Both ganglioneuromas and pheochromocytomas are generally slow growing masses compared with neuroblastomas (19), occurring in middle-aged to older dogs (20), and are both frequently incidental findings during a necropsy (21). Contrary to the other two tumors, neuroblastomas are reported in young dogs, similarly to what is described in humans (8). Histologically, human and dog neuroblastomas share many characteristics which include the presence of small round cells divided into small lobules by a delicate fibrovascular stroma with round deeply staining nuclei and little cytoplasm (22). The adrenal tumor in the present study was positive for NSE, SYN, and neurofilament protein, and negative for vimentin, cytokeratins, and lymphoid cell markers, similar to the characteristics of neuroblastomas in general (7, 8). Franquemont et al. (23), in a comparative study to identify neuroblastomatous foci in composite adrenal pheochromocytoma-neuroblastoma, reported that pheocromocytoma cells, but not neuroblastoma cells, are positive for GFAP, similar to what we observed in this case (23). Moreover, the tumor was positive for NSE, generally considered as a marker for neuroblastomas (8, 18). Although ganglioneuroblastoma was considered in the differential diagnosis, it was disregarded. These tumors are of intermediate malignancy with at least 50% of the differentiated ganglion cells fully matured with abundant cytoplasm and large nuclei with distinct nucleoli (24). Contrary to what was observed in the present case, GFAP and myelin basic protein immunolabeling have been reported, suggesting the presence of non-myelinating and myelinating Schwann cells, respectively (25).

Considering the high proliferation rate revealed by the Ki-67 immunolabeling, a high risk of recurrence rate was expected. The benefit of a combined treatment is unknown in dogs but chemotherapy was suggested based on treatment strategies in humans with neuroblastoma. The aim of adjuvant chemotherapy in children is to control any residual disease (16, 17). However, after the histological results were obtained, the owners unexpectedly declined chemotherapy. Their reasons were related to the time post-surgery, approximately 15 days, the condition of the dog after the surgery, the environmental risks associated with chemotherapy and its possible side effects.

Abdominal and lumbosacral pain was again observed by the owners 33 days after the procedure. Pain associated with tumor progression (metastatic spread or local recurrence) could be of neuropathic origin. The owners were not convinced of the benefits of pursuing further treatments. The animal was therefore euthanized upon their request. Post-mortem findings revealed metastatic spread to the lymph nodes and liver, but not the bone, which could have justified the origin of the perceived pain. Neuroblastoma can metastasize through the lymphatic or hematogenous route, affecting bone, liver, and noncontiguous lymph nodes (1). Similar observations were reported in dogs (2, 8, 9). The quick metastatic spread, in the authors opinion, could be from surgical seeding because manipulation of the retroperitoneal area may have caused some trauma to the visceral organs which created lymphatic or hematologic seeding opportunities. A second explanation could be the presence of undiagnosed metastatic disease at the time of surgery. Although both ultrasound and CT scan were used to stage the disease, slices and algorithms used in the CT series were limited and could have missed the metastasis. Using triple-phase contrast CT and thinner slices could contribute to a more effective screening of metastasis. During surgical inspection, before and after tumor excision, no evidence of metastasis was seen. However, the presence of microscopic disease could not be excluded. Immunohistochemical features pointed to a fast-growing tumor (high Ki-67 labeling index) and this may explain the time frame of development of the macroscopic metastatic disease in the present case.

## Concluding remarks

This is the first report of surgical resection of a large adrenal neuroblastoma in a dog. We hope that this information will help others who may encounter similar cases.

## Author Contributions

LM, MM, SJ, and MP drafted the manuscript, AF critically revised the manuscript, and HP substantially contributed to the study. All authors approved the final version of the manuscript.

## Consent

The patient was treated using the highest standards of care at our veterinary teaching hospital in all steps of diagnosis and treatment. We obtained the consent of the owner of the animal according to the teaching hospital policy.

### Conflict of Interest Statement

The authors declare that the research was conducted in the absence of any commercial or financial relationships that could be construed as a potential conflict of interest.

## Supplementary Material

Supplementary Figure 1 Fine needle aspiration cytology of the abdominal mass.

Supplementary Figure 2 Adrenal tumor. Diffuse and light labeling for non-specific enolase (NSE) (immunohistochemistry using NSE antibody counterstained with Gill2 hematoxylin). Bar = 100 μm.

Supplementary Figure 3 Adrenal tumor. Diffuse and light marking for synaptophysin (immunohistochemistry using synaptophysin antibody counterstained with Gill2 hematoxylin). Bar = 100 μm.

Supplementary Figure 4 Adrenal tumor. Strong marking of 10% of the tumor area for neurofilament protein (immunohistochemistry using neurofilament protein antibody counterstained with Gill2 hematoxylin). Bar = 100 μm.

Supplementary Figure 5 Adrenal tumor. Sixty Eight Percent of the tumor cells were estimated to be positive for Ki67 (immunohistochemistry using Mib-1 antibody counterstained with Gill2 hematoxylin). Bar = 100 μm.

Supplementary Video 1 Contrast enhanced abdominal computed tomography axial images in a movie sequence. Dog, male, 11 month-old, Rhodesian Ridgeback (made in Osirix).
