# Supplementary figures and images for: Surgical Excision of an Adrenal Neuroblastoma in a Dog
Source: Front Vet Sci. 2019 May 29;6:161. doi: 10.3389/fvets.2019.00161 (PMC6555272; doi:10.3389/fvets.2019.00161)

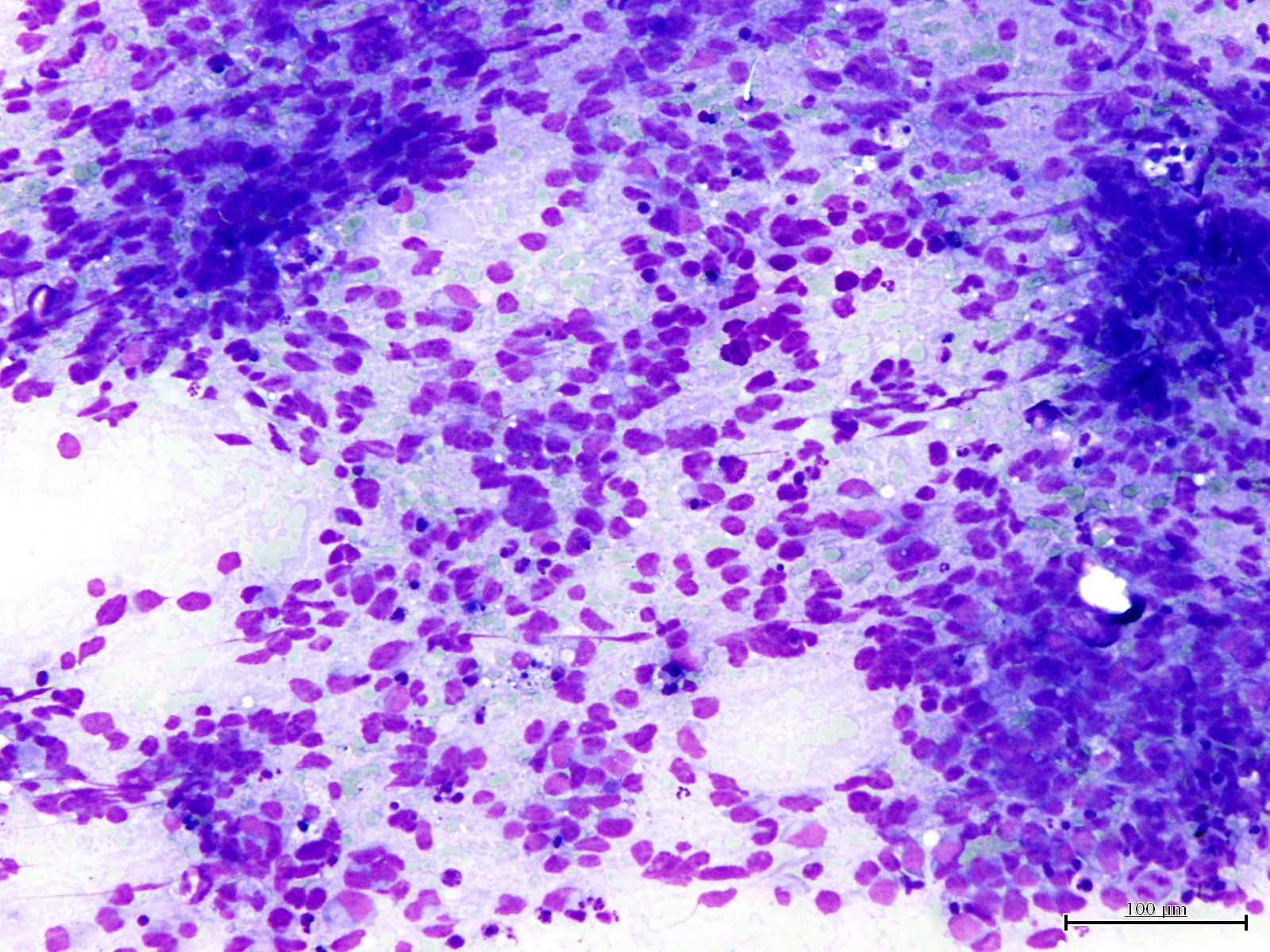

Supplement: Supplementary Figure 1 — Fine needle aspiration cytology of the abdominal mass. [file Image_1.TIF]

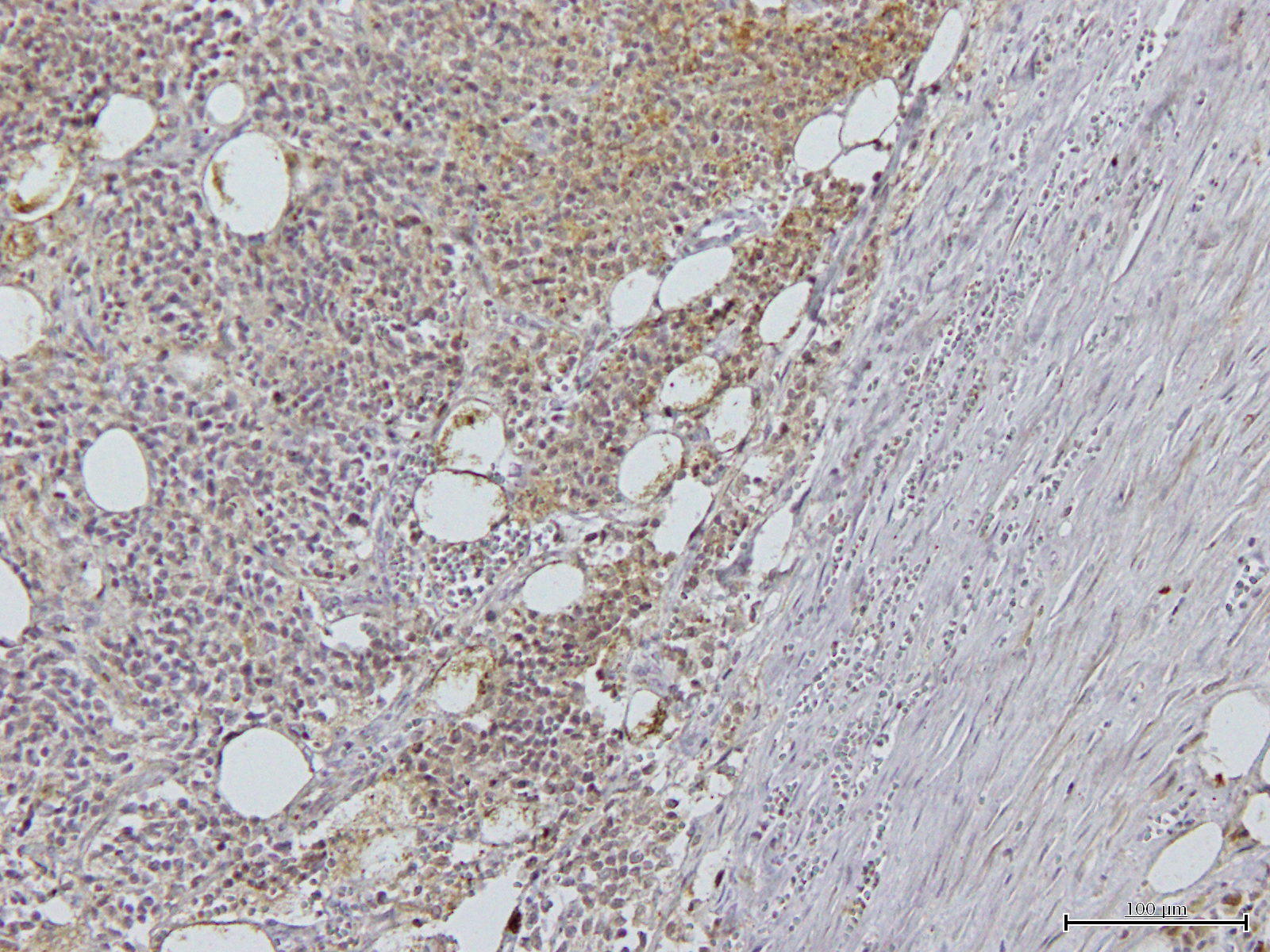

Supplement: Supplementary Figure 2 — Adrenal tumor. Diffuse and light labeling for non-specific enolase (NSE) (immunohistochemistry using NSE antibody counterstained with Gill2 hematoxylin). Bar = 100 μm. [file Image_2.TIF]

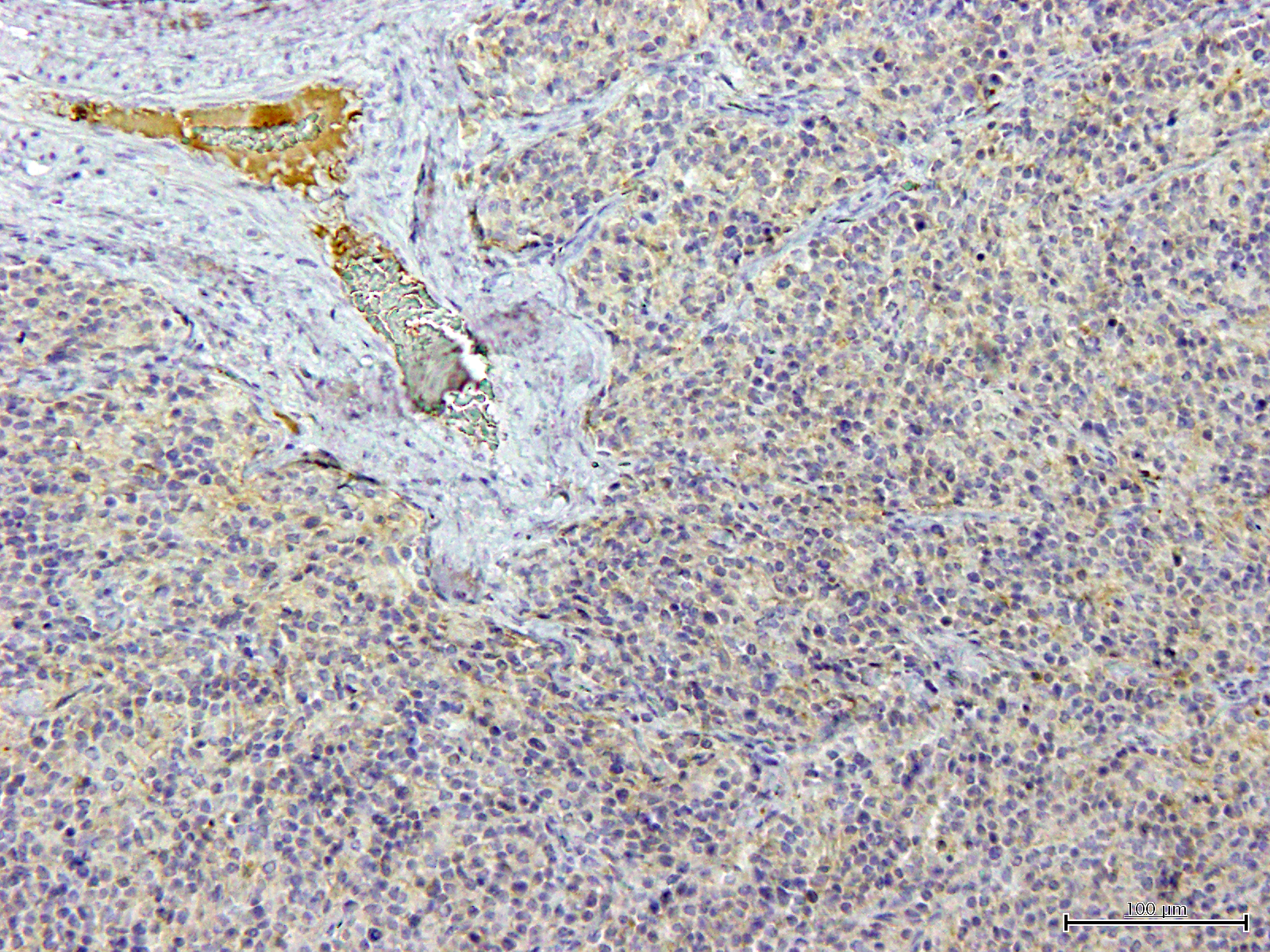

Supplement: Supplementary Figure 3 — Adrenal tumor. Diffuse and light marking for synaptophysin (immunohistochemistry using synaptophysin antibody counterstained with Gill2 hematoxylin). Bar = 100 μm. [file Image_3.TIF]

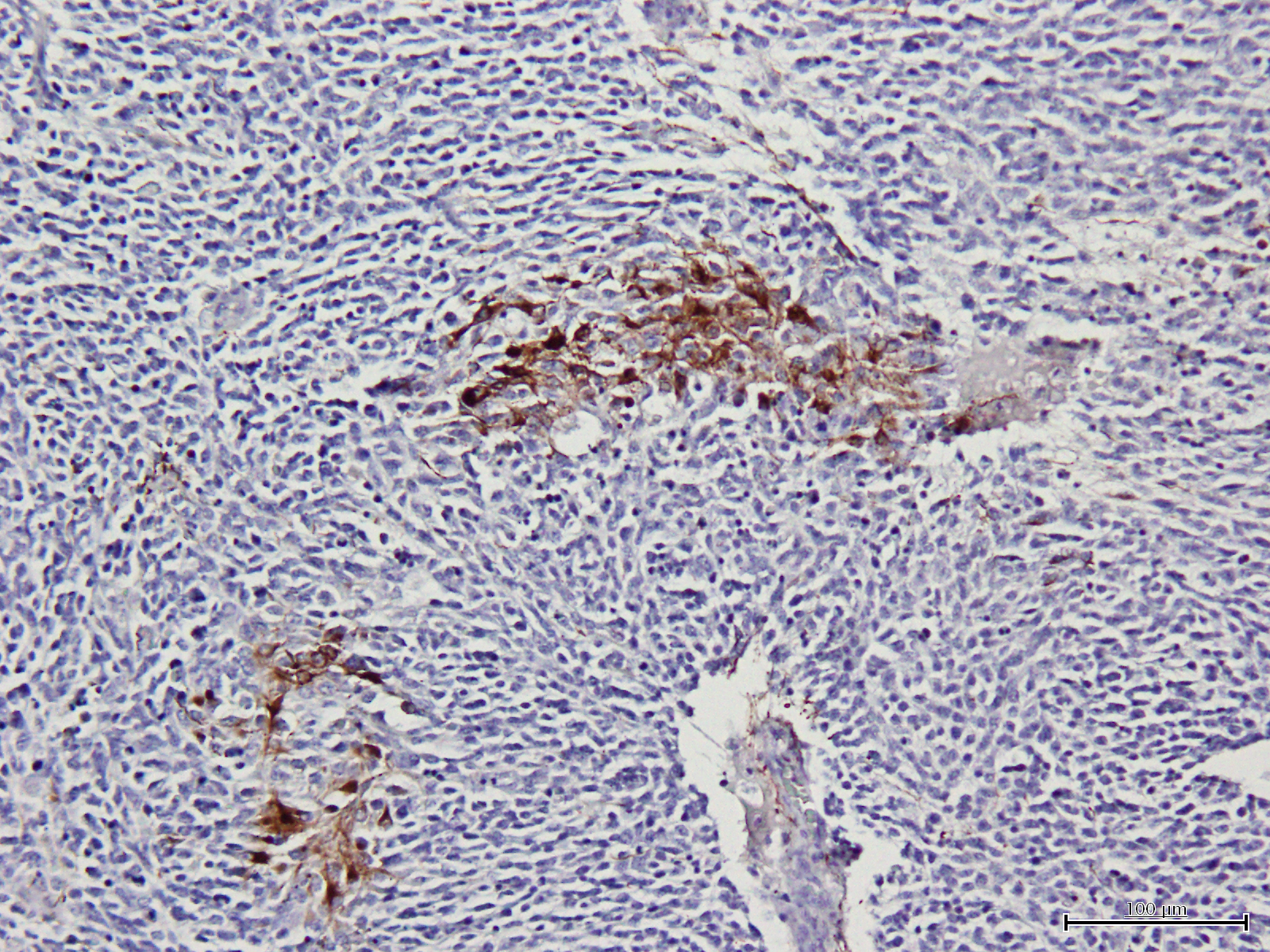

Supplement: Supplementary Figure 4 — Adrenal tumor. Strong marking of 10% of the tumor area for neurofilament protein (immunohistochemistry using neurofilament protein antibody counterstained with Gill2 hematoxylin). Bar = 100 μm. [file Image_4.TIF]

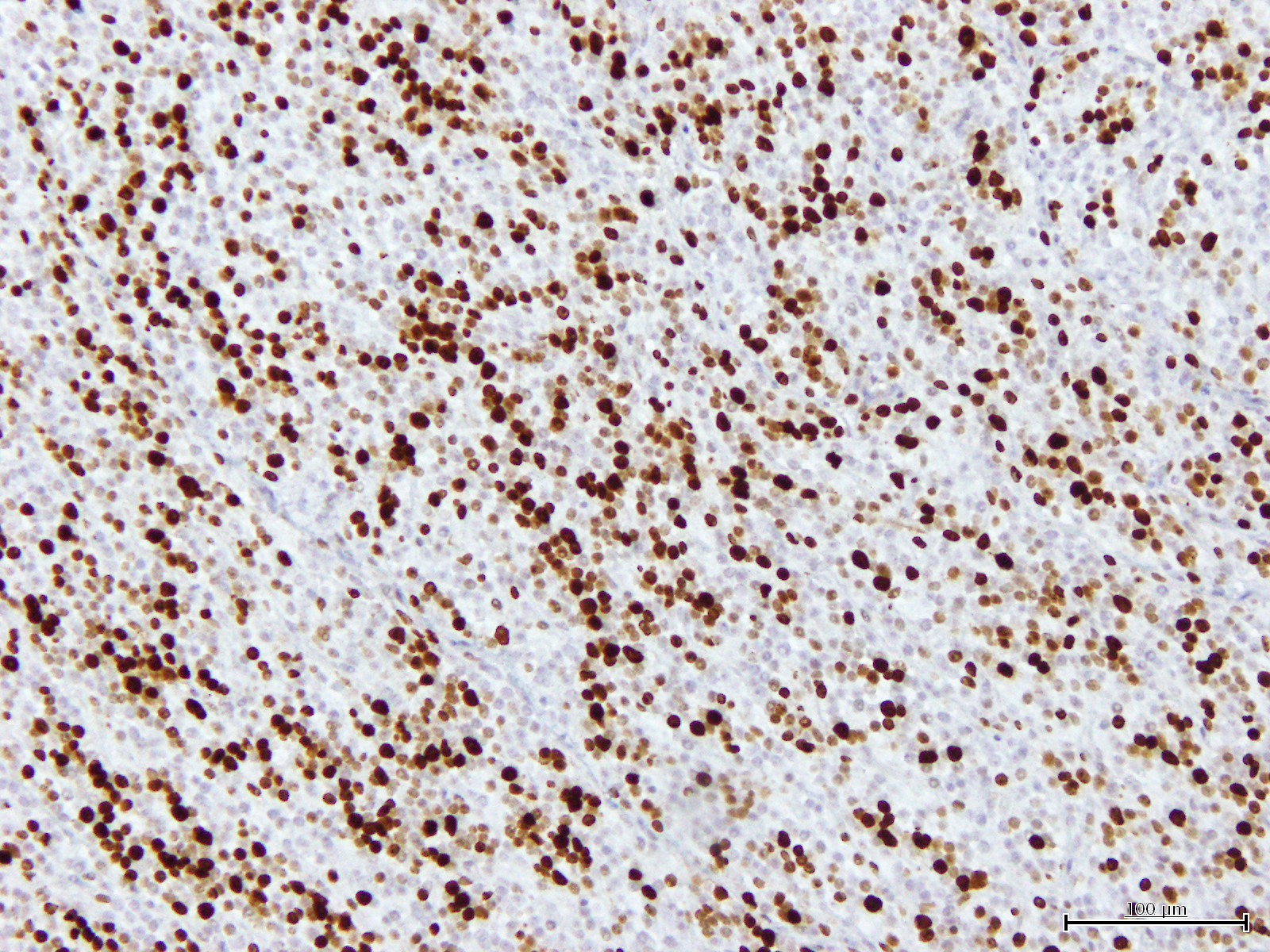

Supplement: Supplementary Figure 5 — Adrenal tumor. Sixty Eight Percent of the tumor cells were estimated to be positive for Ki67 (immunohistochemistry using Mib-1 antibody counterstained with Gill2 hematoxylin). Bar = 100 μm. [file Image_5.TIF]
